# Association of Low Serum Adiponectin Levels with Aortic Arterial Stiffness in Patients with Type 2 Diabetes

**DOI:** 10.3390/jcm8060887

**Published:** 2019-06-21

**Authors:** Cian-Huei Shih, Bang-Gee Hsu, Jia-Sian Hou, Du-An Wu, Yi-Maun Subeq

**Affiliations:** 1 Department of Nursing, Hualien Tzu Chi Hospital, Buddhist Tzu Chi Medical Foundation, Hualien 97004, Taiwan; ivyivy706217@gmail.com; 2 Institute of Medical Sciences, Tzu Chi University, Hualien 97004, Taiwan; gee.lily@msa.hinet.net (B.-G.H.); simianlkive@gmail.com (J.-S.H.); 3 School of Medicine, Tzu Chi University, Hualien 97004, Taiwan; despdu@yahoo.com.tw; 4 Division of Nephrology, Hualien Tzu Chi Hospital, Buddhist Tzu Chi Medical Foundation, Hualien 97004, Taiwan; 5 Division of Metabolism and Endocrinology, Hualien Tzu Chi Hospital, Buddhist Tzu Chi Medical Foundation, Hualien 97004, Taiwan; 6 Department of Nursing, National Taichung University of Science and Technology, Taichung 40401, Taiwan

**Keywords:** adiponectin, aortic arterial stiffness, type 2 diabetes mellitus

## Abstract

Adiponectin, an anti-inflammatory and anti-atherogenic protein, affects glucose metabolism. High serum adiponectin levels are associated with decreased diabetes mellitus (DM) risks. Aortic arterial stiffness (AS) is associated with cardiovascular disease and mortality in type 2 DM patients. We assessed the association between adiponectin levels and aortic AS in type 2 DM patients. We measured serum adiponectin levels in 140 volunteers with type 2 DM and assigned patients with carotid–femoral pulse wave velocity (cfPWV) >10 m/s to the aortic AS group (*n* = 54, 38.6%). These patients had higher systolic (*p* = 0.001) and diastolic (*p* = 0.010) blood pressures; body fat masses (*p* = 0.041); serum triglyceride (*p* = 0.026), phosphorus (*p* = 0.037), and insulin (*p* = 0.040) levels; and homeostasis model assessment of insulin resistance values (*p* = 0.029) and lower estimated glomerular filtration rates (*p* = 0.009) and serum adiponectin levels (*p* = 0.001) than controls. Multivariable logistic regression analysis adjusted for confounders showed serum adiponectin levels (OR 0.922; 95% CI, 0.876–0.970; *p* = 0.002) as an independent predictor of aortic AS. Multivariable forward stepwise linear regression analyses showed that serum adiponectin levels (β = −0.283, adjusted R^2^ change: 0.054, *p* < 0.001) were negatively associated with cfPWV. Thus, serum adiponectin level is an independent predictor of aortic AS in type 2 DM patients.

## 1. Introduction

Adiponectin is an adipocytokine with insulin-sensitizing, anti-atherogenic, and anti-inflammatory properties [1]. It is the most abundant adipocytokine effectively affecting glucose and lipid metabolism [1]. Adiponectin activates the 5’-adenosine monophosphate-activated protein kinase and peroxisome proliferator-activated receptor-α pathways that have insulin-sensitizing effects [2]. Furthermore, adiponectin suppresses oxidative stress and inflammation, thereby improving insulin resistance in type 2 diabetes mellitus (DM) patients [3]. Low adiponectin levels may help predict type 2 DM development, and serum adiponectin levels may enhance insulin sensitivity in DM patients [4]. Moreover, low serum adiponectin levels can be used to predict both future abdominal visceral fat accumulation and increased insulin resistance [5]. Adiponectin deficiency associated with obesity, metabolic syndrome, and type 2 DM may contribute to accelerated atherosclerosis and vascular dysfunction [6]. Low serum adiponectin levels can also be used to predict atherosclerotic cardiovascular diseases and myocardial infarction [7].

Carotid–femoral pulse wave velocity (cfPWV) has been widely accepted in clinical practice as a gold standard for determining central arterial stiffness (AS) [8]. Increased cfPWV values are an early risk marker for DM [9]. Moreover, AS is positively associated with the progression of microvascular complications of DM such as nephropathy, retinopathy, and neuropathy [10]. Indeed, increased AS is a crucial predictor of the development of cardiovascular disease and mortality in type 2 DM patients [11]. Recent studies have suggested that low circulating adiponectin levels are associated with aortic AS in patients undergoing kidney transplantation or hemodialysis, in those with chronic kidney disease, and in young normotensive type 1 DM patients [12,13,14,15]. However, the association between aortic AS and serum adiponectin levels remains unknown in type 2 DM patients. Therefore, this study aimed to evaluate the association between fasting serum adiponectin levels and aortic AS in type 2 DM patients. 

## 2. Experimental Section

### 2.1. Participants

This was a cross-sectional study, and the Protection of the Human Subjects Institutional Review Board of the Tzu Chi University and Hospital approved the study protocol (IRB103-136-B, October 2014). We recruited 140 type 2 DM patients at a medical center in Eastern Taiwan from November 2014 to March 2015. All participants took a 10-min rest and signed their informed consent forms; following this, trained staff measured their blood pressures (BPs) using standard mercury sphygmomanometers with appropriate cuff sizes. We analyzed averaged values of participants’ systolic BP (SBP) and diastolic BP (DBP), derived from three measurements at 5-min intervals. We included type 2 DM patients aged >18 years and excluded those with malignancies, acute infections, heart failure, or acute myocardial infarctions and those who refused to provide informed consent for the study.

### 2.2. Anthropometric Analysis

Patients’ waist circumferences were measured using a measuring tape around the waist from the point between the lowest ribs and the hip bones. Body weights were measured, with patients in light clothing and without shoes, to the nearest 0.5 kg and body heights to the nearest 0.5 cm. Body mass indexes (BMIs) were calculated as weight in kilograms divided by height in squared meters. We used a single-frequency (50 kHz) analyzer to measure whole-body bioimpedances (hand–foot) of the fat mass on the basis of the standard tetrapolar (Biodynamic-450, Biodynamics Corporation, Seattle, USA). Same operator obtained all the measurements [12,13,16,17].

### 2.3. Biochemical Investigations

All participants’ fasting blood samples (approximately 5 mL) were immediately centrifuged at 3000 *g* for 10 min. We used an autoanalyzer to measure the serum levels of fasting glucose, total calcium, phosphorous, glycated hemoglobin (HbA1c), blood urea nitrogen (BUN), creatinine, triglycerides (TGs), total cholesterol (TCH), low-density lipoprotein cholesterol (LDL-C), and high-density lipoprotein cholesterol (HDL-C) (Siemens Advia 1800, Siemens Healthcare GmbH, Henkestr, Germany) [12,13,16,17]. Random spot urine testing was performed for determining urine albumin-to-creatinine ratios (UACRs). Insulin (Labor Diagnostika Nord, Nordhorn, Germany) and serum total adiponectin (SPI-BIO, Montigny le Bretonneux, France) levels were measured using commercially available enzyme-linked immunosorbent assays [12,13]. We used the Chronic Kidney Disease Epidemiology Collaboration equation to estimate glomerular filtration rates (eGFRs). We calculated the homeostatic model assessment of insulin resistance (HOMA-IR) value to quantify insulin resistance as follows: HOMA-IR = fasting plasma glucose (mg/dL) × fasting serum insulin (μU/mL)/405 [18].

### 2.4. Aortic Stiffness According to Carotid–Femoral Pulse Wave Velocity Measurements

We applied pressure applanation tonometry (SphygmoCor system, AtCor Medical, Australia) to measure aortic AS according to cfPWV values [15,16]. Briefly, we asked patients to rest for 10 min in a quiet and temperature-controlled room and then obtained measurements with the participants in the supine position. We obtained cfPWV measurements at two superficial artery sites (the carotid–femoral segment) and provided an *R*-timing reference by recording electrocardiogram signals. We then used an integral software to calculate the mean time difference between *R*-wave and pulse wave on a beat-to-beat basis, with an average of 10 consecutive cardiac cycles. We assigned patients with cfPWV values > 10 m/s to the aortic AS group [16,17,19].

### 2.5. Statistical Analysis

We tested data using Kolmogorov–Smirnov test and expressed normally distributed data as means ± standard deviations tested using two-tailed independent t-tests. Non-normally distributed data were expressed as medians and interquartile ranges and tested using Mann–Whitney U tests. We analyzed the number of patients using χ^2^ tests. Because data on age; TG, fasting glucose, HbA1c, BUN, creatinine, adiponectin, and insulin levels; UACR; and HOMA-IR values were non-normally distributed, we transformed them logarithmically to achieve normality. Clinical variables that correlated with cfPWV values in type 2 DM patients were evaluated using simple linear regression analyses, followed by multivariable forward stepwise regression analyses. We used multivariable logistic regression analyses to test variables that were significantly associated with aortic AS (independent predictors). All data were analyzed using the SPSS for Windows (version 19.0; SPSS, Chicago, IL, USA), and all *p* values <0.05 were considered significant.

## 3. Results

Table 1 presents the clinical characteristics of 140 type 2 DM patients; of them, 54 (38.6%) patients were assigned in the aortic AS group. These patients had significantly lower serum adiponectin levels (*p* = 0.001) and eGFR (*p* = 0.009), and higher body fat mass (*p* = 0.041), SBP (*p* = 0.001), DBP (*p* = 0.010), TG levels (*p* = 0.026), UACR (*p* = 0.030), insulin levels (*p* = 0.040), and HOMA-IR value (*p* = 0.029) than those in the control group. The mean values for number of males; number of comorbid conditions with hypertension; and number of patients using antihypertensive, anti-lipid, and anti-diabetic drugs were similar between the two groups.

Multivariable logistic regression analyses performed to determine the factors significantly associated with aortic AS showed that serum adiponectin levels (odds ratio (OR), 0.922; 95% confidence interval (CI), 0.876–0.970; *p* = 0.002) were the only independent predictor of aortic AS in type 2 DM patients (Table 2).

Table 3 displays the simple and multivariable regression analyses of the correlation between cfPWV and clinical variables among the type 2 DM patients. Simple regression analyses showed that cfPWV values were positively correlated with logarithmically transformed age (log-age, *r* = 0.230; *p* = 0.006), waist circumference (*r* = 0.218; *p* = 0.010), body fat mass (*r* = 0.236; *p* = 0.005), SBP (*r* = 0.339; *p* < 0.001), DBP (*r* = 0.276; *p* = 0.001), log-TG (*r* = 0.249; *p* = 0.003), log-creatinine (*r* = 0.204; *p* = 0.016), log-UACR (*r* = 0.186; *p* = 0.028), log-insulin (*r* = 0.212; *p* = 0.012), and log-HOMA-IR (*r* = 0.217; *p* = 0.010), while they were negatively correlated with eGFR (*r* = −0.263; *p* = 0.002), and serum adiponectin levels (*r* = −0.296; *p* < 0.001). In multivariable forward stepwise linear regression analyses, log-age (β = 0.220; adjusted R^2^ change, 0.040; *p* = 0.005) and SBP (β = 0.254; adjusted R^2^ change, 0.109; *p* = 0.002) were positively associated, whereas serum adiponectin levels (β = −0.283; adjusted R^2^ change, 0.054; *p* < 0.001) were negatively associated with cfPWV in type 2 DM patients.

## 4. Discussion

Our results suggest that log-age and SBP are positively associated and serum log-adiponectin levels are negatively associated with cfPWV in type 2 DM patients. Moreover, low serum adiponectin levels are positively associated with aortic AS in type 2 DM patients after adjusting for significant confounders.

AS, measured according to cfPWV values, has been found to be a cardiovascular risk marker independent of the traditional risk factors for atherosclerosis [8]. In type 2 DM patients, each 1 m/s increase in the aortic PWV is associated with a hazard ratio increase of 8% in all-cause and cardiovascular mortalities [11]. In aging people, the vasculature undergoes structural and functional changes that are characterized by endothelial dysfunction, vascular wall thickening, reduced vascular distensibility, and increased arterial stiffening [20]. Moreover, aging-associated vascular changes are accelerated by arterial hypertension [20]. Elevated BP promotes vascular extracellular matrix synthesis and increases vascular structure stiffening and thickness as well as AS [21]. Our results showed that type 2 DM patients with AS had significantly higher SBP and DBP and were older than those without AS, and that SBP and age are positively associated with cfPWV in type 2 DM patients.

Increased body fat mass may promote adverse vascular changes, such as AS [22]. Excess body fat mass and low fat-free mass are associated with AS in type 2 DM patients [23]. Increased TG levels were found to be significantly associated with increased cfPWV in a community-based, prospective study [24]. Insulin resistance, a primary biochemical abnormality in type 2 DM patients, increases AS and is an important risk factor for subclinical atherosclerosis [25]. Zhang et al. [26] have reported that increased cfPWV at baseline predicts future albuminuria progression in type 2 DM patients. In the Chronic Renal Insufficiency Cohort study, decreased eGFR was found to be independently associated with an increase in cfPWV [27]. Our study also showed that type 2 DM patients with AS had higher body fat mass, serum TG and insulin levels, HOMA-IR value, and UACRs and lower eGFR than controls.

Adiponectin functions as an insulin sensitizer and exhibits antidiabetic effects [1,2,3]. It exerts potent antioxidant effects on human vascular walls by reducing the enzymatic activity of NADPH oxidase (NOX)1 and NOX2 isoforms, decreasing the generation of superoxide and increasing the bioavailability of tetrahydrobiopterin. Adiponectin improves endothelial nitric oxide synthase (eNOS) coupling and increases nitric oxide (NO) bioavailability by activating the phosphatidylinositol 3-kinase/Akt pathway on human vascular walls [28]. Adiponectin exhibits anti-inflammatory effects that inhibit nuclear factor-kappa B activation in vascular endothelial cells [29]. Decreased adiponectin expression results in decreased NO production, increased vascular inflammation, and insulin resistance; all these factors are known to promote atherosclerosis [30]. Adiponectin may act as an endogenous modulator of vascular remodeling, enhancing NO production, activating eNOS, and decreasing AS by reducing inflammatory mediators, neointimal thickening, and vascular smooth muscle cell proliferation [6,31]. In this study, after adjusting for various confounders in the multivariable forward stepwise linear regression analysis, we found that serum adiponectin levels were negatively correlated with cfPWV in type 2 DM patients; moreover, our multivariable logistic regression analysis revealed that decreased serum adiponectin levels were an independent predictor of aortic AS in these patients.

The strength of this study is that, to the best of our knowledge, it is the first study to report a negative correlation between serum adiponectin levels and central AS in type 2 DM patients. We are aware of the limitations of this study. First, this was a cross-sectional study with a limited sample size. Second, the participants were recruited from a single medical center in Taiwan. Third, circulating adiponectin has multiple isoforms, of which high-molecular-weight adiponectin is thought to be the most powerful of biologic effects. However, there is no gold standard for distinguishing between adiponectin isoform levels [32]. Finally, many prescription drugs used by the patients have been reported to affect AS, such as oral hypoglycemic agents, antihypertensive drugs, and statins [33]. However, no significant associations between these drugs and AS have been found. Further related studies are suggested to confirm the association between serum adiponectin levels and aortic AS in type 2 DM patients.

## 5. Conclusions

This study revealed that age and SBP are positively associated and serum adiponectin levels are negatively associated with cfPWV in type 2 DM patients; moreover, low serum adiponectin levels are positively associated with aortic AS among these patients.

## Figures and Tables

**Table 1:** Clinical variables of the 140 type 2 diabetes mellitus patients in the high aortic stiffness and control groups.

Characteristics	All Patients (*n* = 140)	Control Group (*n* = 86)	Aortic Stiffness Group (*n* = 54)	*p* Value
Age (years)	62.69 (58.00–70.75)	61.55 (56.75–68.00)	64.52 (58.00–74.25)	0.086
Height (cm)	161.98 ± 8.33	162.52 ± 8.29	161.13 ± 8.38	0.338
Body weight (kg)	70.02 ± 12.73	69.86 ± 12.99	70.28 ± 12.41	0.848
Waist circumference (cm)	90.25 ± 8.96	89.51 ± 9.05	91.44 ± 8.78	0.218
Body mass index (kg/m^2^)	26.59 ± 3.76	26.35 ± 3.75	26.97 ± 3.77	0.344
Body fat mass (%)	30.94 ± 7.64	29.90 ± 7.60	32.60 ± 7.48	0.041 *
cfPWV (m/s)	9.73 ± 2.64	8.13 ± 1.38	12.28 ± 2.13	<0.001 *
SBP (mmHg)	140.49 ± 18.89	136.40 ± 17.01	147.00 ± 20.04	0.001 *
DBP (mmHg)	82.03 ± 10.59	80.21 ± 9.36	84.93 ± 11.81	0.010 *
Total cholesterol (mg/dL)	160.84 ± 30.15	161.33 ± 27.31	160.06 ± 34.44	0.809
Triglyceride (mg/dL)	135.59 (83.25–170.50)	129.24 (72.00–153.50)	145.70 (94.75–189.50)	0.026 *
HDL-C (mg/dL)	47.11 ± 13.04	48.58 ± 12.30	44.78 ± 13.93	0.093
LDL-C (mg/dL)	98.06 ± 25.87	98.26 ± 24.12	97.74 ± 28.66	0.909
Fasting glucose (mg/dL)	152.99 (121.00–175.00)	149.73 (118.75–172.25)	158.17 (121.00–183.50)	0.401
Glycated hemoglobin (%)	7.80 (6.60–8.78)	7.76 (6.50–8.80)	7.87 (6.60–8.78)	0.447
Blood urea nitrogen (mg/dL)	17.21 (12.00–19.00)	16.65 (12.00–18.00)	18.11 (13.75–20.25)	0.142
Creatinine (mg/dL)	0.91 (0.70–1.00)	0.87 (0.70–1.00)	0.97 (0.80–1.20)	0.093
eGFR (mL/min)	87.22 ± 26.90	91.87 ± 27.15	79.82 ± 24.99	0.009 *
UACR (mg/g)	14.34 (7.20–53.99)	11.50 (7.10–31.10)	25.23 (8.44–164.55)	0.030 *
Total calcium (mg/dL)	9.07 ± 0.44	9.10 ± 0.45	9.05 ± 0.39	0.468
Phosphorus (mg/dL)	3.60 ± 0.49	3.54 ± 0.48	3.69 ± 0.51	0.071
Insulin (uIU/mL)	10.11 (3.54–12.03)	9.21 (2.92–10.83)	11.54 (4.46–15.04)	0.040 *
HOMA-IR	2.22 (1.10–4.21)	2.03 (0.89–3.63)	2.65 (1.48–5.55)	0.029 *
Adiponectin (μg/mL)	28.92 (23.41–36.56)	31.30 (24.96–40.38)	20.01 (20.47–33.23)	0.001 *
Male, *n* (%)	58 (41.4)	33 (38.4)	25 (46.3)	0.354
Hypertension, *n* (%)	76 (54.3)	46 (53.5)	30 (55.6)	0.811
ACE inhibitor use, *n* (%)	9 (6.4)	6 (7.0)	3 (5.6)	0.739
ARB use, *n* (%)	54 (38.6)	30 (34.9)	24 (44.4)	0.258
β-blocker use, *n* (%)	20 (14.3)	10 (11.6)	10 (18.5)	0.257
CCB use, *n* (%)	44 (31.4)	27 (31.4)	17 (31.5)	0.991
Statin use, *n* (%)	66 (47.1)	41 (47.7)	25 (46.3)	0.874
Fibrate use, *n* (%)	8 (5.7)	5 (5.8)	3 (5.6)	0.949
Metformin use, *n* (%)	76 (54.3)	48 (55.8)	28 (51.9)	0.647
Sulfonylureas use, *n* (%)	77 (55.0)	46 (53.5)	31 (57.4)	0.650
DDP-4 inhibitor use, *n* (%)	87 (62.1)	55 (64.0)	32 (59.3)	0.577
Insulin use, *n* (%)	38 (27.1)	23 (26.7)	15 (27.8)	0.894

Values for continuous variables are expressed as means ± standard deviations and tested by Student’s t-test; non-normally distributed variables are expressed as medians and interquartile ranges and tested by Mann–Whitney U test; values are presented as numbers (%) and analyses were done using the chi-square test. AS, arterial stiffness; cfPWV, carotid–femoral pulse wave velocity; SBP, systolic blood pressure; DBP, diastolic blood pressure; HDL-C, high-density lipoprotein cholesterol; LDL-C, low-density lipoprotein cholesterol; eGFR, estimated glomerular filtration rate; HOMA-IR, homeostasis model assessment of insulin resistance; ACE, angiotensin-converting enzyme; ARB, angiotensin receptor blocker; CCB, calcium channel blocker; DDP-4, dipeptidyl peptidase 4. * *p* < 0.05 was considered statistically significant.

**Table 2:** Multivariable logistic regression analysis of the factors associated with aortic stiffness among the 140 type 2 diabetic mellitus patients.

Variables	Odds Ratio	95% Confidence Interval	*p* Value
Adiponectin (μg/mL)	0.922	0.876–0.970	0.002 *
Age (years)	1.022	0.9782–1.067	0.339
Body fat mass (%)	1.030	0.976–1.086	0.279
Systolic blood pressure (mmHg)	1.018	0.985–1.052	0.288
Diastolic blood pressure (mmHg)	1.003	0.947–1.063	0.912
Estimated glomerular filtration rate (mL/min)	0.990	0.972–1.010	0.324
Urine albumin-to-creatinine ratio (mg/g)	1.000	1.000–1.001	0.2212
Triglyceride (mg/dL)	0.999	0.993–1.005	0.716
Insulin (uIU/mL)	0.946	0.854–1.048	0.290
HOMA-IR	1.095	0.877–1.367	0.424

Data analysis was done using the multivariable logistic regression analysis (adopted factors: age, body fat mass, systolic blood pressure, diastolic blood pressure, triglyceride levels, estimated glomerular filtration rate, urine albumin-to-creatinine ratio, insulin levels, HOMA-IR, and adiponectin level). HOMA-IR, homeostasis model assessment of insulin resistance. * *p* < 0.05 was considered statistically significant.

**Table 3:** Correlation between carotid–femoral pulse wave velocity and clinical variables among type 2 diabetes mellitus patients.

Variables	Carotid–femoral Pulse Wave Velocity (m/s)				
	Simple Linear Regression		Multivariable Linear Regression		
	*r*	*p* Value	Beta	Adjusted R^2^ Change	*p* Value
Female	0.043	0.615	-	-	-
Hypertension	0.151	0.075	-	-	-
Log-Age (years)	0.230	0.006 *	0.220	0.040	0.005 *
Height (cm)	−0.070	0.412	-	-	-
Body weight (kg)	0.067	0.433	-	-	-
Waist circumference (cm)	0.218	0.010 *	-	-	-
Body mass index (kg/m^2^)	0.139	0.101	-	-	-
Body fat mass (%)	0.236	0.005 *	-	-	-
Systolic blood pressure (mmHg)	0.339	<0.001 *	0.254	0.109	0.002 *
Diastolic blood pressure (mmHg)	0.276	0.001 *	-	-	-
Total cholesterol (mg/dL)	−0.029	0.733	-	-	-
Log-Triglyceride (mg/dL)	0.249	0.003 *	-	-	-
HDL-C (mg/dL)	−0.124	0.145	-	-	-
LDL-C (mg/dL)	−0.083	0.327	-	-	-
Log-Glucose (mg/dL)	0.084	0.322	-	-	-
Log-Glycated hemoglobin (%)	0.071	0.402	-	-	-
Log-BUN (mg/dL)	0.126	0.139	-	-	-
Log-Creatinine (mg/dL)	0.204	0.016 *	-	-	-
eGFR (mL/min)	−0.263	0.002 *	-	-	-
Log-UACR (mg/g)	0.186	0.028 *	-	-	-
Total calcium (mg/dL)	−0.085	0.322	-	-	-
Phosphorus (mg/dL)	0.026	0.763	-	-	-
Log-Insulin (uIU/mL)	0.212	0.012 *	-	-	-
Log-HOMA-IR	0.217	0.010 *	-	-	-
Log-Adiponectin (μg/mL)	−0.296	<0.001 *	−0.283	0.054	<0.001 *

Data on age, triglycerides, glucose, HbA1c, BUN, creatinine, UACR, insulin, HOMA-IR and adiponectin levels showed skewed distributions and were log-transformed before the analyses. Analysis of data was done using simple linear regression or multivariable stepwise linear regression analyses (adapted factors included log-age, waist circumference, body fat mass, SBP, DBP, log-Triglyceride, eGFR, log-UACR, log-insulin, log-HOMA-IR, and log-adiponectin). HDL-C, high-density lipoprotein cholesterol; LDL-C, low-density lipoprotein cholesterol; BUN, blood urea nitrogen; eGFR, estimated glomerular filtration rate; UACR, urine albumin-to-creatinine ratio; HOMA-IR, homeostasis model assessment of insulin resistance. * *p* < 0.05 was considered statistically significant.

## References

[1] Adamczak M., Więcek A. (2013). The Adipose Tissue as an Endocrine Organ. Semin. Nephrol..

[2] Kim Y., Park C.W. (2019). Mechanisms of Adiponectin Action: Implication of Adiponectin Receptor Agonism in Diabetic Kidney Disease. Int. J. Mol. Sci..

[3] Yanai H., Yoshida H. (2019). Beneficial Effects of Adiponectin on Glucose and Lipid Metabolism and Atherosclerotic Progression: Mechanisms and Perspectives. Int. J. Mol. Sci..

[4] Von Frankenberg A.D., Reis A.F., Gerchman F. (2017). Relationships between adiponectin levels, the metabolic syndrome, and type 2 diabetes: a literature review. Arch. Endocrinol. Metab..

[5] Han S.J., Boyko E.J., Fujimoto W.Y., Kahn S.E., Leonetti D.L. (2017). Low Plasma Adiponectin Concentrations Predict Increases in Visceral Adiposity and Insulin Resistance. J. Clin. Endocrinol. Metab..

[6] El Husseny M.W.A., Mamdouh M., Shaban S., Abushouk A.I., Zaki M.M.M., Ahmed O.M., Abdel-Daim M.M. (2017). Adipokines: Potential Therapeutic Targets for Vascular Dysfunction in Type II Diabetes Mellitus and Obesity. J. Diabetes Res..

[7] Kishida K., Funahashi T., Shimomura I. (2014). Adiponectin as a routine clinical biomarker. Best Pr. Res. Clin. Endocrinol. Metab..

[8] Vlachopoulos C., Xaplanteris P., Aboyans V., Brodmann M., Cifkova R., Cosentino F., De Carlo M., Gallino A., Landmesser U., Laurent S. (2015). The role of vascular biomarkers for primary and secondary prevention. A position paper from the european society of cardiology working group on peripheral circulation: Endorsed by the association for research into arterial structure and physiology (artery) society. Atherosclerosis.

[9] Muhammad I.F., Borné Y., Östling G., Kennbäck C., Gottsäter M., Persson M., Nilsson P.M., Engström G. (2017). Arterial Stiffness and Incidence of Diabetes: A Population-Based Cohort Study. Diabetes Care.

[10] Prenner S.B., Chirinos J.A. (2015). Arterial stiffness in diabetes mellitus. Atheroscler..

[11] Cruickshank K., Riste L., Anderson S.G., Wright J.S., Dunn G., Gosling R.G. (2002). Aortic pulse-wave velocity and its relationship to mortality in diabetes and glucose intolerance: an integrated index of vascular function?. Circulation.

[12] Ho C.-C., Hsu B.-G., Yin W.-Y., Ho G.-J., Chen Y.-C., Lee M.-C. (2016). Serum adiponectin is a negative predictor of central arterial stiffness in kidney transplant patients. Scand. J. Clin. Lab. Investig..

[13] Hou J.-S., Wang C.-H., Lai Y.-H., Lin Y.-L., Kuo C.-H., Subeq Y.-M., Hsu B.-G. (2018). Negative Correlation of Serum Adiponectin Levels With Carotid–Femoral Pulse Wave Velocity in Patients Treated With Hemodialysis. Boil. Res. Nurs..

[14] Kim C.S., Bae E.H., Ma S.K., Park S.K., Lee J.Y., Chung W., Lee K., Kim Y.H., Oh K.H., Ahn C. (2017). Association of serum adiponectin concentration with aortic arterial stiffness in chronic kidney disease: From the KNOW-CKD study. Clin. Exp. Nephrol..

[15] Tsiakou A., Liatis S., Alexiadou K., Diakoumopoulou E., Makrilakis K., Tentolouris N., Kyriaki D., Katsilambros N. (2013). Arterial Stiffness Is Inversely Related to Plasma Adiponectin Levels in Young Normotensive Patients With Type 1 Diabetes. Diabetes Care.

[16] Su I.-M., Wu D.-A., Lee C.-J., Hou J.-S., Hsu B.-G., Wang J.-H. (2018). Serum cystatin C is independently associated with aortic arterial stiffness in patients with type 2 diabetes. Clin. Chim. Acta.

[17] Tseng P.W., Hou J.S., Wu D.A., Hsu B.G. (2019). High serum adipocyte fatty acid binding protein concentration linked with increased aortic arterial stiffness in patients with type 2 diabetes. Clin. Chim. Acta.

[18] Li J.-C., Wu D.-A., Hou J.-S., Subeq Y.-M., Chen H.-D., Hsu B.-G. (2016). High Serum Adipocyte Fatty Acid Binding Protein Is Associated with Metabolic Syndrome in Patients with Type 2 Diabetes. J. Diabetes Res..

[19] Mancia G., Fagard R., Narkiewicz K., Redon J., Zanchetti A., Bohm M., Christiaens T., Cifkova R., De Backer G., Dominiczak A. (2013). 2013 ESH/ESC guidelines for the management of arterial hypertension: The task force for the management of arterial hypertension of the European society of hypertension (ESH) and of the european society of cardiology (ESC). J. Hypertens..

[20] Harvey A., Montezano A.C., Lopes R.A., Ríos F., Touyz R.M. (2016). Vascular Fibrosis in Aging and Hypertension: Molecular Mechanisms and Clinical Implications. Can. J. Cardiol..

[21] Safar M.E., Asmar R., Benetos A., Blacher J., Boutouyrie P., Lacolley P., Laurent S., London G., Pannier B., Protogerou A. (2018). Interaction Between Hypertension and Arterial Stiffness. Hypertens..

[22] Lim S., Meigs J.B. (2013). Ectopic fat and cardiometabolic and vascular risk. Int. J. Cardiol..

[23] Anoop S., Misra A., Bhardwaj S., Gulati S. (2015). High body fat and low muscle mass are associated with increased arterial stiffness in Asian Indians in North India. J. Diabetes its Complicat..

[24] Wang X., Ye P., Cao R., Yang X., Xiao W., Zhang Y., Bai Y., Wu H. (2016). Triglycerides are a predictive factor for arterial stiffness: a community-based 4.8-year prospective study. Lipids Heal. Dis..

[25] Kozàkovà M., Palombo C., Ojo O. (2016). Diabetes Mellitus, Arterial Wall, and Cardiovascular Risk Assessment. Int. J. Environ. Res. Public Heal..

[26] Zhang X., Low S., Sum C.F., Tavintharan S., Yeoh L.Y., Liu J., Li N., Ang K., Lee S.B., Tang W.E. (2017). Arterial stiffness is an independent predictor for albuminuria progression among Asians with type 2 diabetes—A prospective cohort study. J. Diabetes its Complicat..

[27] Townsend R.R., Wimmer N.J., Chirinos J.A., Parsa A., Weir M., Perumal K., Lash J.P., Chen J., Steigerwalt S.P., Flack J. (2010). Aortic PWV in chronic kidney disease: A CRIC ancillary study. Am. J. Hypertens..

[28] Woodward L., Akoumianakis I., Antoniades C. (2017). Unravelling the adiponectin paradox: novel roles of adiponectin in the regulation of cardiovascular disease. Br. J. Pharmacol..

[29] Parker-Duffen J.L., Walsh K. (2014). Cardiometabolic effects of adiponectin. Best Pr. Res. Clin. Endocrinol. Metab..

[30] Shibata R., Ouchi N., Ohashi K., Murohara T. (2017). The role of adipokines in cardiovascular disease. J. Cardiol..

[31] Sabbatini A.R., Fontana V., Laurent S., Moreno H. (2015). An update on the role of adipokines in arterial stiffness and hypertension. J. Hypertens..

[32] Van Andel M., Heijboer A.C., Drent M.L. (2018). Adiponectin and Its Isoforms in Pathophysiology. Advances in Clinical Chemistry.

[33] Hussein A.M., Shaaya G., Arora R., Al-Khazaali A., Al-Khafaji K., Helu H.K. (2016). Therapeutic Modulation of Aortic Stiffness. Am. J. Ther..

